# The role of MRI and Ultrasonography in Diagnosis and Treatment of Glenohumeral Joint Adhesive Capsulitis

**DOI:** 10.31138/mjr.34.1.7

**Published:** 2023-03-31

**Authors:** Madalena Pimenta, Evangelia E. Vassalou, Beatriz Cardoso-Marinho, Michail E. Klontzas, Sofia Dimitri-Pinheiro, Apostolos H. Karantanas

**Affiliations:** 1 Department of Radiology, Armed Forces Hospital, D. Pedro V Universitary Clinical Centre, Porto, Portugal,; 2 Hospital Cuf Porto, Porto, Portugal,; 3 Faculdade de Medicina, Porto, Portugal,; 4 Department of Radiology, General District Hospital, Sitia, Greece,; 5 Portugal Football School, Portuguese Football Federation, Oeiras, Portugal,; 6 Research Center in Sports Sciences, CIDESD, University of Maia, Portugal,; 7 Portuguese Institute of Sports and Youth, IPDJ, Sports Medicine Center, Porto, Portugal,; 8 Department of Radiology, School of Medicine, University of Crete, Heraklion, Greece,; 9 Department of Medical Imaging, University Hospital, Heraklion, Greece,; 10 Foundation for Research and Technology Hellas (FORTH), Computational Biomedicine Laboratory (CBML), Heraklion, Greece,; 11 Biomedicine Department, Faculty of Medicine, University of Porto, Porto, and I3S-Institute for Innovation and Health Research, University of Porto, Porto, Portugal

**Keywords:** frozen shoulder, adhesive capsulitis, MR imaging, ultrasonography, hydrodilatation, treatment/ultrasound-guided

## Abstract

Adhesive capsulitis is a common disorder of the glenohumeral joint. Delayed diagnosis is the result of overlapping signs and symptoms with other disorders affecting the shoulder. Typically, the disease shows gradual progression of pain and loss of the range of motion. The hallmark of the physical examination is limitation of both passive and active motion without any associated degenerative changes on plain radiographs. Conservative and/or surgical treatments have shown conflicting results. Poor outcome may be related to co-morbid factors mainly including prolonged immobilization, rotator cuff pathology and diabetes mellitus among others. This review will present the current literature data on the natural history and pathophysiology of the disease, and will highlight the role of imaging in the prompt and accurate diagnosis as well as in the imaged-guided treatment with emphasis on ultrasonography.

## INTRODUCTION

Adhesive capsulitis (AC) is a painful and disabling disorder with progressive pain and disability associated with limitation of passive and active range of motion (ROM) of the glenohumeral joint (GHJ).^1^ The terms “frozen shoulder”, introduced by Codman in 1934, and adhesive capsulitis (AC), proposed by Neviaser in 1945, have been interchangeably used to describe the condition.^2^ The entity is defined by the American Academy of Orthopaedic Surgeons as “a condition of varying severity characterized by the gradual development of global limitation of active and passive shoulder motion where radiographic findings other than osteopenia are absent”.^3^ Although pathologic studies have shown constant abnormalities, the cause of the disease remains unclear. Currently, it is accepted that AC represents the end-stage of an inflammatory process resulting in GHJ capsular contraction typically involving the rotator cuff interval (RCI) and the containing coracohumeral ligament (CHL), subsequently leading to restricted range of motion (ROM).^4^ AC is a clinical diagnosis made on the basis of medical history and physical examination.^5^ Exclusion of any other cause of overlapping symptoms is essential before the diagnosis of AC is rendered. In equivocal cases, imaging is indicated to rule out other causes of a painful and stiff shoulder and for confirming the clinical suspicion.^6,7^

## EPIDEMIOLOGY AND PATHOPHYSIOLOGY

AC of the GHJ has a prevalence of 2-5% in the general population, increasing up to 30% in insulin-dependent diabetic patients with the highest incidence in women aged 40-60 years.^8,9^ Lundberg classified AC into primary or idiopathic when a definite cause or inciting event could not be identified and secondary when the condition is associated with pre-existing pathologies.^10^ Prolonged immobilization, recent shoulder surgery, dislocation, rotator cuff tendinopathy, calcific tendinitis or osteoarthritis, Dupuytren’s disease, hypothyroidism, obesity, stroke, breast cancer treatment, cardiopulmonary disease, hyperlipidaemia, myocardial infarction and autoimmune diseases have been reported to predispose to AC; however, the association between most of these conditions and AC has yet to be defined.^11^ Diabetes in particular, has been linked to more protracted and difficult AC clinical course, with diabetic patients showing worse functional outcomes compared to their non-diabetic counterparts.^12^ Additionally, higher predilection of AC was found in white patients, patients with a positive family history and HLA-B27 positivity, suggesting a genetic predisposition.^13^

Although the precise pathogenesis of AC remains unclear, it is currently accepted that the development of the condition involves an interplay between inflammatory and fibrotic processes, primarily affecting the GHJ capsule and particularly the RCI, a structure that is critical to GHJ stability, with the containing CHL.^4,14^ Pathogenetically, altered levels of matrix metalloproteinases and their tissue inhibitors have been identified in patients with AC, potentially implying an imbalance between fibrogenetic processes, tissue remodelling and degradation as a pathogenetic insult.^15,16^ Additionally, there is evidence that elevated levels of cytokines, mainly including (IL)-1a and -1b, tumour necrosis factor (TNF)-a, cyclooxygenase (COX)-1 and -2, in the articular and peri-articular tissues of patients with AC are involved in an inflammatory process leading to fibrotic changes.^15,17^ These inflammatory factors facilitate tissue repair and remodelling and appear to be predominately involved in the cellular mechanisms of sustained inflammation and fibrosis, mostly in primary AC.^15^ Although the initial stimulus is unknown, it has been suggested that a minor insult could initiate an inflammatory healing response leading to excess accumulation and propagation of fibroblasts releasing type I and type III collagen.^15^ Moreover, the over-expression of vascular and neural markers in tissues from shoulders with AC, suggest that both neoangiogenesis and neoinnervation may have a vital role in the pathogenetic pathway.^18^

## NATURAL HISTORY AND CLINICAL PRESENTATION

Although AC is usually characterized by a self-limiting course within a period of 12-18 months, 20% - 60% of patients may experience enduring symptoms and functional disability, lasting up to 7 years.^19–22^

There are four clinical stages of adhesive capsulitis based on the clinical, arthroscopic and histologic findings.^4,8^ In stage 1 or pre-adhesive stage, lasting up to 3 months, there is mild hypervascular and inflammatory synovitis and the patients present with mild end-range pain, deteriorating at night and not significantly responding to non-steroidal anti-inflammatory (NSAIDs) and analgesic drugs. ROM is generally preserved. During this stage, a misdiagnosis of rotator cuff impingement and/or subacromial bursitis is not unusual. In stage 2 or “freezing” stage, with a mean duration of symptoms between 3 and 9 months, there is synovial proliferation and the patients frequently complain of intense pain near the end-range of movement. In stage 3 or “frozen” stage, synovitis gradually resolves but mature adhesions develop due to the proliferation of by dense collagenous tissue within the capsule. The duration of symptoms ranges between 4 and 12 months and is denoted by profound limitation of ROM with low grade pain. Severe capsular retraction without apparent synovitis defines stage 4, the “thawing” phase, with a duration between 12 and 42 months. Patients typically present with painless stiffness and gradually improved shoulder mobility.

The pain in AC is often poorly localised or located in the anterior or posterior aspect of the shoulder, with/-out radiation along the biceps tendon. The pain is constant and severe presenting typically at rest, with a strong night-time component, often preventing lying on the affected side. As the disease progresses, the pain typically downscales, but severely decreased ROM caused by thickening and contraction of the joint capsule and synovial lining persist. The clinical diagnosis is based on the following criteria: painful and stiff shoulder lasting for at least 4 weeks, severe shoulder pain that interferes with daily activities, nocturnal pain, painful restriction of both active and passive elevation to less than 100°, 50% restriction of external rotation as compared to the normal contralateral side and normal plain radiographs.^23^ The clinical diagnosis shows high sensitivity and specificity, provided that the signs and symptoms are typical and the disease is well established.^24^

The differential diagnosis in the absence of typical clinical features includes acromioclavicular arthropathy, subacromial-subdeltoid bursitis, inflammatory or degenerative glenohumeral arthropathy, biceps tendinopathy, rotator cuff disease, and cervical disc herniation.^4,24^

## IMAGING

Early diagnosis and treatment of AC can lead to markedly improved clinical management. Diagnosis is usually based on physical examination alone. However, imaging studies can clarify the diagnosis in equivocal cases showing overlapping symptoms with other shoulder pathologies.^4^

**Figure 1.**
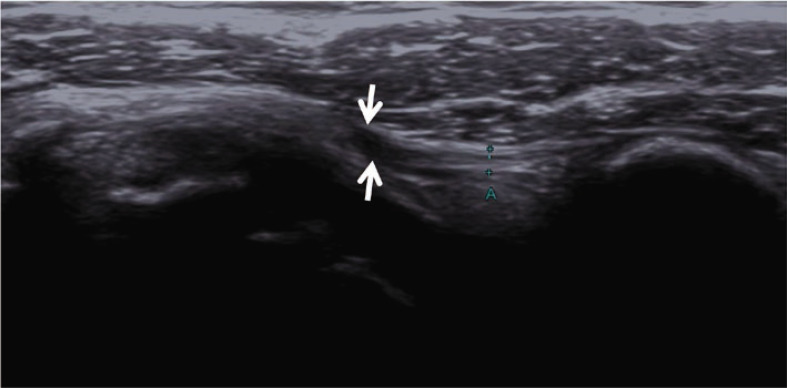
Oblique US image along the coracohumeral ligament in a 79-year-old male patient with a clinical diagnosis of adhesive capsulitis. Thickening of the ligament is shown (arrows).

**Figure 2.**
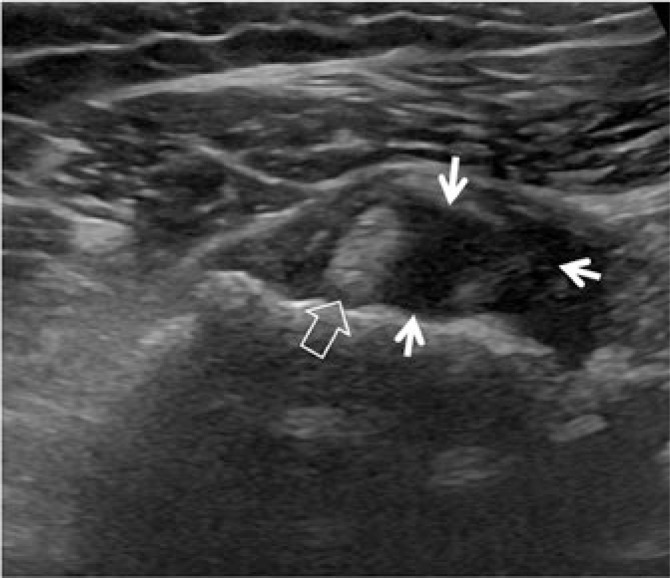
Oblique sagittal US image shows an abnormal hypoechoic tissue in the rotator cuff interval (arrows). The long head biceps tendon is also shown (open arrow).

Conventional arthrography reveals limited GHJ capacity and a small or missing axillary recess.^1,25^ Due to its inability to directly assess the synovium and capsule, the method is not currently widely used. CT-arthrography seems to be reliable when limited GHJ capacity is confirmed at injection. Decreased width of the axillary recess, thickening of the lateral and to a lesser extent of the medial capsular wall, are demonstrated.^26^ The value of ultrasonography (US) in the diagnosis of AC remains not well defined. Supporting to this, AC was not included among clinical indications for musculoskeletal US suggested by the European Society of Musculoskeletal Radiology.^27^ However, several recent reports support that the method may provide hints towards the diagnosis of the condition. The major US findings in patients with AC is thickening of the CHL and presence of hypoechoic soft tissue and increased Doppler vascular flow in the RCI resulting from hypervascular scar tissue (**Figures 1 and 2**).^28–30^ The fibrovascular inflammatory soft tissue changes in the RCI, have been reported to occur up to 100% of patients.^28^ Axillary recess capsular thickening and Doppler signal in the RCI, may often support the diagnosis. Studies have showed that a cut-off value of 2.2 mm for the CHL and 4 mm for the axillary recess show good clinical correlation with AC.^31^ Others suggest that CHL thickness of more than 1.2 mm should be regarded as abnormal.^30^ Application of shear wave elastography, has showed that the CHL is stiffer in patients with AC.^32^ Another useful sign during passive external rotation, is reduced sliding of the infraspinatus tendon with folding towards the joint capsule changing the tendon’s shape from flat to concave.^33^ As a rule, no significant joint effusion is shown on either US or MR imaging.

**Figure 3.**
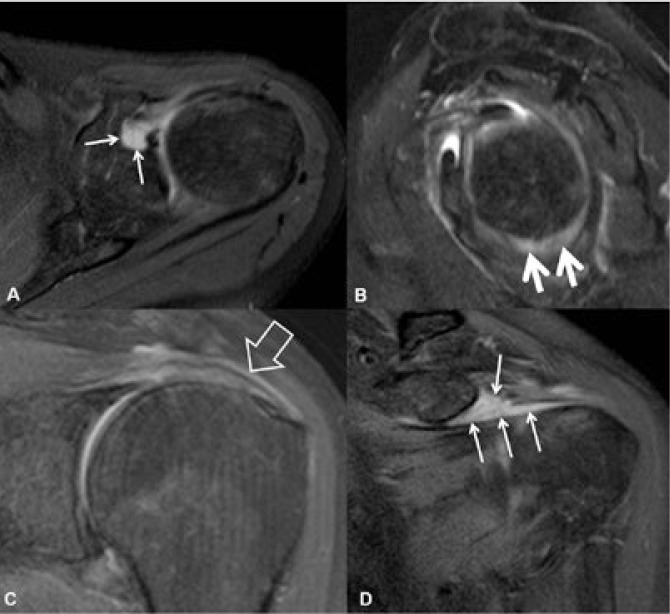
A 62-year-old female patient with 2-month pain in the left shoulder. Fat suppressed PD-w MR images in the axial **(a)**, oblique sagittal **(b)**, and oblique coronal **(c,d)** planes, show the abnormal signal in the rotator cuff interval (thin arrows **a,d**), and in the axillary recess (thick arrows, **b**). The abnormal signal in the supraspinatus tendon (open arrow, **c**), suggests degenerative tendinopathy.

**Figure 4.**
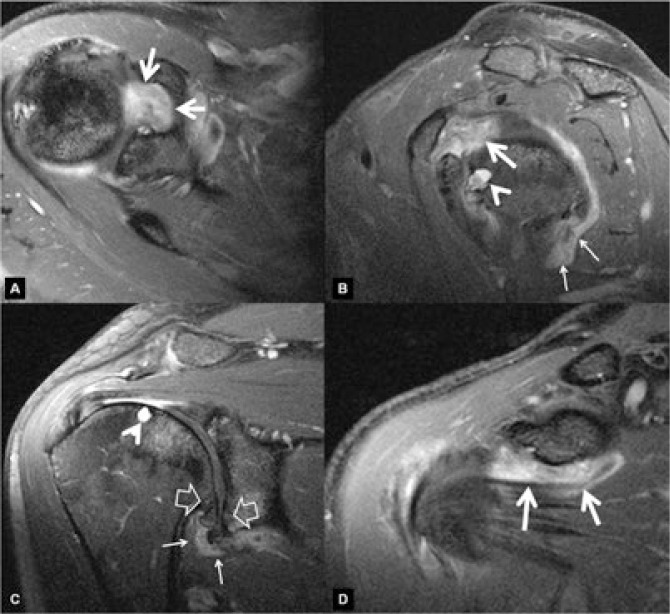
A 66-year-old female patient with a history of CPPD and osteoarthritis in the right shoulder. Fat suppressed contrast enhanced T1-w MR images in the axial **(a)**, oblique sagittal **(b)** and oblique coronal **(c,d)** planes, show the abnormal enhancement in the rotator cuff interval (thick arrows **a,b,d**), and in the axillary recess (thin arrows, **b,c**). Crystal induced osteoarthritis is shown in the lower glenohumeral joint with osteophyte formation (open arrows, **c**). A small subcortical degenerative cyst is shown in the humeral head (arrowheads **b,c**).

**Figure 5.**
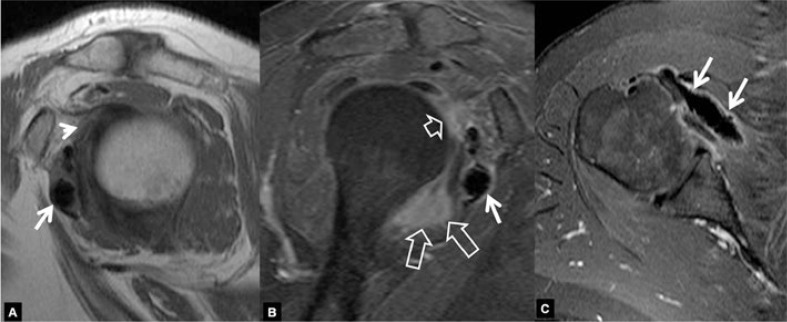
A 58-year-old female patient with a history of hydroxyapatite deposition disease over the subscapularis tendon and second ray adhesive capsulitis. The oblique sagittal T1-w **(a)** and fat suppressed contrast enhanced T1-w oblique sagittal **(b)** and axial **(c)** MR images show the calcific tendinopathy (arrows, **a,b,c**), the abnormal signal in the rotator cuff interval (arrowhead **a**) and the enhancement located in the rotator cuff interval (short open arrow) and in the axillary recess (long open arrows).

MR imaging has been used as plain, enhanced, or by utilizing direct or indirect arthrography. On plain MR imaging, a capsular thickening of more than 4 mm in the axillary recess was a useful criterion for the diagnosis of AC (**Figure 3**).^34^ A common finding is the depiction of a poorly defined soft tissue lesion encasing the CHL (**Figure 4**).^35^ Fat suppressed T2-w images alone, often show increased signal at the inferior glenohumeral ligament with a high sensitivity and sensitivity for AC diagnosis.^36^ An extracapsular hyperintense layer bordering the outer capsular surface and the inferior glenohumeral ligament was suggested to be highly specific (up to 97%).^36^ Another study utilising fat suppressed T2-w images, showed correlation between the extracapsular oedema and the location of symptoms, as well as an association with the clinical stage of the disease.^37^ Abnormal soft tissue in the RCI was associated with variable degree of enhancement in in almost all patients, surrounding the biceps anchor in the majority and in axillary pouch in 40% of them (**Figures 3–5**).^35,38,39^ Anterior capsular thickening and abnormal hyperintensity on T2-w images, has been recently described as being a useful sign for diagnosis.^40^

**Figure 6.**
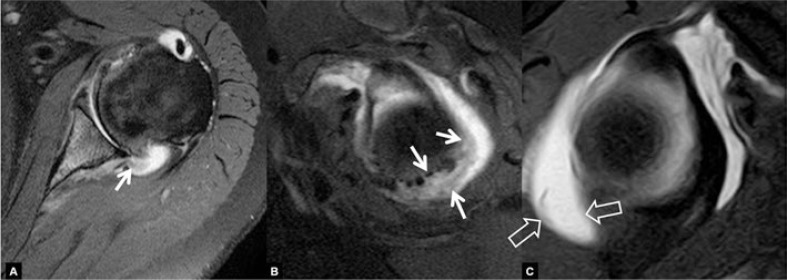
MR arthrographic fat suppressed T1-w MR images in the axial **(a)** and oblique sagittal **(b,c)** planes in a patient with a diagnosis of adhesive capsulitis **(a,b)**, there is abnormal tissue in the inferior and posterior axillary recess (arrows) and a limited capacity of the joint which is not distended. In a patient with shoulder instability without any indication of adhesive capsulitis **(c)**, a normal joint space configuration is shown (open arrows).

On MR arthrography, useful diagnostic criteria for the diagnosis of AC include thickening, more than 4mm, of the joint capsule and synovium as well as reduced width and height of the axillary recess (**Figure 6**).^41,42^ Variable results have been shown by other studies where no significant differences existed between patients and controls.^43,44^ Taking into account that the RCI and the CHL represent key structures involved in AC, it was shown that the RCI dimensions are reduced in patients as opposed in controls.^41,43,45^ Indirect arthrography findings suggestive of AC, include thickening of the joint capsule in the axillary recess and enhancement of the axillary recess and the RCI.^46^ In our practice, we do not apply regularly neither direct nor indirect MR arthrography. Plain MR imaging matched with a good clinical examination, can accurately establish the diagnosis with better accuracy as compared with clinical evaluation alone or matched with US. Contrast-enhanced MR imaging is helpful in clinically difficult cases with coexistent abnormalities.^47^

## TREATMENT OPTIONS

The treatment of AC is perhaps the most controversial aspect of this disorder. Several options have been proposed, but the evidence is still poor.^48^ Broadly, treatment strategies include conservative, minimally invasive or surgical approaches.^49^ The treatment is related directly to the stage of the disease and aims to reduce pain and restore the GHJ ROM.^50^ In stage II or freezing phase, with pain being the predominant complaint, steroid injection plays an important role. In stage III or frozen phase, treatment aims in increasing the ROM of the GHJ, primarily with physiotherapy. In stage 4 or thawing phase, scapular function should be addressed with rehabilitation due to its long-standing compensatory role in the course of the disease.

### Conservative treatment

Initial treatment consists of oral NSAIDs and analgesic drugs combined with physiotherapy. The latter, aims to prevent further limitation of shoulder mobility and to restore the ROM.^51^ Overall, there is little evidence to support physical therapy alone in the treatment of AC.^52^ NSAIDs are generally recommended, as an adjunct to physical therapy, for short-term pain relief during the early inflammatory stages of the disease.^53^ Pharmacological treatment, when long lasting, may be complicated with gastrointestinal bleeding and renal function impairment. Intra-articular injections of steroids are indicated when oral treatment and physiotherapy fail, and show better results in shoulder disability scores, ROM improvement and patient satisfaction.^54–57^

### Minimal invasive treatment

#### a. Steroid and Sodium hyaluronate injection

Intra-articularly administered steroids are commonly used to provide symptoms management, often in combination with physical therapy, and have been suggested to offer better symptoms improvement compared to oral steroid treatment.^55^ According newly released guidelines, US-guidance improves the outcome of GHJ injections compared to palpation-guided or sham injections in adhesive capsulitis up to 12 weeks.^58,59^ Various combinations of treatments have been utilized with contradictive results. Combined injection with lidocaine and hyaluronic acid shows promising results regarding pain relief and improvement of ROM.^60^ According to a systematic review, sodium hyaluronate injection is associated with improved ROM, constant scores and pain relief in the short-term.^61^ According to another study, the addition of hyaluronate injected into the GHJ has not been proved superior to physical therapy.^62^ A recent review showed that an intra-articular injection of 20-40mg of triamcinolone, resulted in pain relief for a period of up to 24 weeks, without established long-term effect.^63^ It has also been shown that subacromial injection has equal efficacy.^63^ Caution is required in patients on protease inhibitors as triamcinolone injections may result in adrenal suppression and Cushing syndrome. Regarding the concern on chondrolysis complicating intra-articular injection of steroids, it does not appear to occur in the context of treating AC.^64^ Hyaluronic injection into the joint could be a good alternative to steroids.

#### b. Hydrodistension

**Figure 7.**
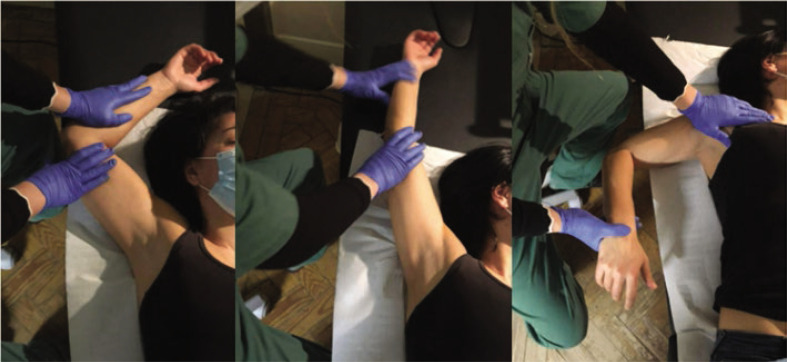
Passive manipulations following hydrodistension, in the supine position.

**Figure 8.**
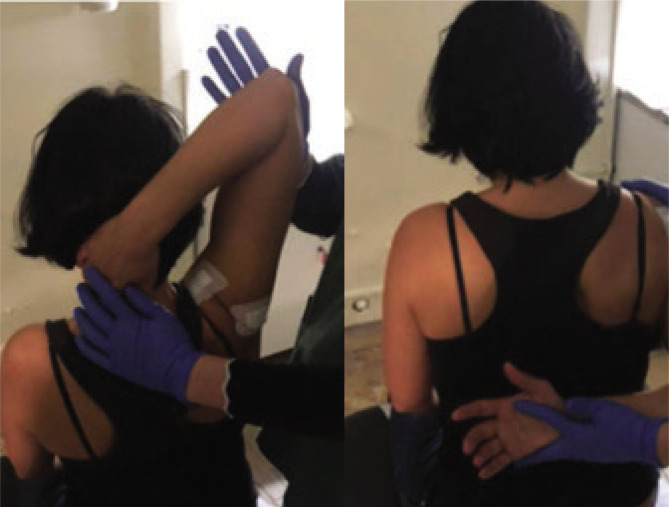
Passive manipulations in the sitting position, following hydrodistension.

For the last two decades, a high-volume saline injection, also known as hydrodilatation or hydrodistension, has been applied with good results. This has been combined with various schemes, ie, with steroids, with capsular rupture, with bursal injection, with hyaluronic acid injection and with repeated injections for second or 3d time. Although historically this was done to the point of capsular rupture, capsule preservation improves short-term outcomes.^65^ A randomised controlled trial in primary care, showed that in long term, 12 months, there was no difference between a) steroid injection with lidocaine, b) steroid injection with lidocaine and distension with saline and c) conservative treatment including physiotherapy, pain killers and per oral steroids medication.^66^ In a recent review based on six randomized studies, showed that combining hydrodilatation with corticosteroid injection, potentially expedites recovery of pain-free ROM.^67^ In our practice, image-guided hydrodistension with steroid injection, with or without bursal injection followed by immediate manipulations or physiotherapy are performed. Manipulations include flexor, extensor, abductor, internal, and external rotator muscle group passive ROM and stretching exercises in the supine and sitting position with a pain tolerance level (**Figures 7 and 8**). US guidance is always recommended because blind injection shows a high failure rate.^68,69^ Depending upon local preferences, hydrodistension can be achieved with fluoroscopy and CT.

#### c. Surgical procedures

Manipulation under general anaesthesia has been for years the second choice following failure of conservative and/or minimal invasive treatments, with a restoration of the ROM in about 80% of patients.^56,70^ This treatment allows a controlled rupture of the capsular contractions, being less invasive as compared to arthroscopic release. However, this treatment is associated with many iatrogenic intraarticular injuries including labral, tendon and ligamentous tears as proved with subsequent arthroscopy and rarely with humeral fractures, rotator cuff tears, glenohumeral dislocation, and injuries to the axillary and radial nerves.^71,72^ A prospective randomized trial showed that manipulation under anaesthesia did not show better results on the 2-year follow up as compared with steroid injection with distension.^73^

Arthroscopic adhesion and capsular release is an established treatment option.^56,74,75^ In the turn of the century, it was used in AC cases which were refractory to closed manipulation. Its limitations though include the invasive procedure, high cost and complications related to the stiffness of the GHJ which increases the risk of chondral injury during insertion of the arthroscope and the risk of axillary nerve injury during inferior capsular release. In addition, surgery may be contraindicated in elderly patients with co-morbidities.

## CONCLUSIONS

Adhesive capsulitis is a common, painful, and disabling disorder that is usually initially managed with a combination of analgesics, steroids, and physiotherapy. The diagnosis is based on clinical grounds. In equivocal cases, the role of imaging investigation is vital. US-guided treatment with steroid injection and hydrodistension, combined with post-procedural physiotherapy, is a promising treatment choice in patients with persisting symptoms.
